# Study protocol of a non-randomized controlled trial on a circumplex model-based motivational training program for pre-service physical education teachers

**DOI:** 10.3389/fpubh.2025.1611556

**Published:** 2025-07-07

**Authors:** Carlos Mayo-Rota, Ángel Abós, Javier García-Cazorla, Zilia Villafaña-Samper, Luis García-González

**Affiliations:** Department of Didactics of the Musical, Plastic and Corporal Expression, Faculty of Health and Sports Sciences, University of Zaragoza, Huesca, Spain

**Keywords:** Self-Determination Theory, (de)motivating teaching styles, Physical Education Teacher Education, quasi-experimental study, mixed-methods research

## Abstract

Physical Education Teacher Education is crucial, as it directly influences how pre-service Physical Education teachers will teach, motivate, and engage their future students. However, training programs that foster motivating teaching styles while minimizing demotivating ones remain scarce, particularly during initial teacher education. This study presents the protocol for a non-randomized controlled trial evaluating a motivational-based training program for pre-service Physical Education teachers, grounded in Self-Determination Theory and the circumplex model. The program, embedded in a Master’s in Physical Education Teacher Education, consists of theoretical and practical training to foster motivating teaching styles and minimize demotivating ones. The study will involve at least 38 pre-service teachers, divided into an experimental group (*n* = 19) and a control group (*n* = 19). The experimental group will participate in a 14-hour training program combining theoretical and practical components. The control group will follow the standard Physical Education Teacher Education curriculum. A quasi-experimental pre-post design with a mixed-methods approach will be used. Quantitative assessments will measure changes in perceived competence, motivation for teaching, and (de)motivating teaching styles, while qualitative focus groups will provide in-depth insights into participants’ experiences and program applicability. To ensure objectivity, independent researchers will conduct assessments, and external experts will moderate the focus groups. Findings will contribute empirical evidence on the effectiveness of Self-Determination Theory-based interventions in initial teacher education, informing curriculum development and supporting the advancement of evidence-based pedagogical training in Physical Education.

**Clinical trial registration:**

ClinicalTrials.gov, identifier NCT06924554.

## Introduction

Teaching begins with learning, and what is learned inevitably shapes both what and how it is taught (1). In this regard, Physical Education Teacher Education (PETE) programs play a critical role in preparing future teachers by equipping them with theoretical knowledge and practical competencies (2, 3). Among these competencies, one of the most crucial is the ability to adopt a motivating teaching style, which refers to how teachers structure their lessons, interact and behave with their students, and create learning environments that foster the quality of students’ motivation (4). Recently, the appearance of the circumplex model (5, 6), grounded in Self-Determination Theory (SDT) (7), provides a comprehensive framework for understanding the various (de)motivating teaching styles (i.e., autonomy support, structure, control, and chaos) that shape physical education (PE) teachers’ pedagogical practices. These styles significantly influence students’ motivational processes, leading to both positive and negative outcomes in PE (8, 9).

Although various SDT-based training programs have been developed to enhance motivating teaching styles among in-service PE teachers (10), research on similar interventions for pre-service teachers remains scarce (11–13). In particular, there is a lack of studies integrating the circumplex model to conceptualize and refine (de)motivating teaching approaches during initial teacher education. Addressing this gap, the present protocol study aims to design a teacher training program grounded in SDT and the circumplex model, embedded within PETE, designed to foster motivating teaching styles while reducing demotivating ones.

According to SDT (7), PE teachers play a pivotal role in shaping students’ motivation through the influence that their (de)motivating teaching styles and approaches have on students’ basic psychological needs (BPN). These teaching styles and approaches can either support or thwart students’ BPN (9). These needs include autonomy (i.e., the perception of being the origin of one’s actions), competence (i.e., feeling effective in interactions and tasks required by the context), and relatedness (i.e., the sense of connection with significant others) (7). To better understand how these styles manifest in PE settings, the SDT-based circumplex model (5, 14) offers a structured framework that classifies teaching styles along two intersecting axes: a vertical axis representing high versus low directiveness (exercised by the teacher in interaction with their students), and a horizontal axis reflecting the extent to which teachers either support or thwart students’ BPN. The intersection of these axes delineates four primary (de)motivating teaching styles (i.e., autonomy support, structure, control, and chaos), each one further subdivided into two specific approaches (i.e., participative, attuning, guiding, clarifying, demanding, domineering, abandoning, awaiting).

The autonomy-supportive style (i.e., need-supportive and low directiveness) encourages students to take the initiative and assume responsibility for their learning. This style incorporates a participative approach, allowing students to make meaningful decisions regarding their learning processes, and/or an attuning approach, which aligns tasks with students’ interests while emphasizing their relevance (5, 14). In addition, the structuring style (i.e., need-supportive and high directiveness) emphasizes guidance and supporting the learning process to enhance students’ sense of competence. This style includes a guiding approach, characterized by the provision of constructive feedback and instructional support, and/or a clarifying approach, which ensures that students clearly understand the learning goals and expectations (5, 14).

In contrast, the controlling style (i.e., low need support and high directiveness) exerts internal and external pressures on students to think, act, or perform in specific ways. This style is associated with a demanding approach, which relies on sanctions, coercion, or extrinsic incentives, and/or a domineering approach, which induces feelings of guilt, shame, or anxiety (5, 14). Finally, the chaotic style (i.e., low need support and low directiveness) reflects a lack of structured guidance and an indifferent teaching attitude. This style is characterized by an abandoning approach, where teachers neglect their students and delegate full responsibility for their learning, and/or an awaiting approach, marked by a lack of planning, granting excessive freedom, and passively observing outcomes (5, 14).

In their teaching, PE teachers do not adopt a single isolated teaching style, as they often combine various (de)motivating teaching styles (15–20). Teachers who predominantly use need-supportive styles (i.e., autonomy support and structure/competence support) while minimizing need-thwarting styles (i.e., control and chaos) tend to promote greater need satisfaction (and lower need frustration), higher autonomous motivation, and lower controlled motivation and amotivation among their students. These outcomes directly enhance students’ learning, enjoyment of PE lessons, and intentions to engage in physical activity outside school (9, 21). Conversely, teachers who rely primarily on controlling and chaotic teaching styles (with low levels of need-supportive ones) tend to produce the opposite effects, leading to lower need satisfaction and autonomous motivation, coupled with higher need frustration, controlled motivation, and amotivation among students, ultimately resulting in lower engagement, enjoyment, learning outcomes, and intentions to be physically active (9). Additionally, some teachers blend autonomy-supportive and structuring approaches with controlling strategies, which can lead to students’ motivational cost in both the short and long term (e.g., lower need satisfaction and less self-determined motivation) (15–20). While research often examines the effects of these teaching styles independently, in real educational settings, teachers frequently apply them in varying degrees rather than as fixed categories. A PE teacher may, for example, predominantly use autonomy-supportive strategies but occasionally resort to controlling styles in response to specific student behaviors or classroom dynamics. This fluidity highlights the importance of not only fostering motivating teaching styles but also systematically reducing demotivating styles. Recognizing the profound benefits associated with need-supportive teaching styles, there has been a recent increase in the implementation of SDT-based training programs designed to enhance PE teachers’ motivating teaching styles. However, research on how to effectively reduce the use of demotivating styles remains scarce, although teachers may adopt both motivating and demotivating styles within the same instructional setting (20). Future interventions should, therefore, not only emphasize the promotion of need-supportive styles but also include targeted strategies to help teachers identify and unlearn demotivating styles, ultimately guiding them toward the best possible teaching profile.

SDT posits that the satisfaction or frustration of BPN (i.e., autonomy, competence, and relatedness) determines an individual’s psychological and motivational development (7, 22). This framework extends across various contexts, including the professional development of PE teachers (23). Additionally, SDT conceptualizes motivation along a self-determination continuum, influencing how individuals pursue and develop their professional roles (22). At the highly self-determined end of this continuum lies autonomous motivation, which encompasses intrinsic motivation (e.g., teaching PE for the inherent enjoyment it brings) and identified regulation (e.g., teaching PE due to its perceived value for students and personal development). As self-determination decreases, controlled motivation emerges, characterized by introjected regulation (e.g., teaching PE to avoid feelings of guilt or enhance self-esteem) and external regulation (e.g., teaching PE in exchange for external rewards such as salary or vacation benefits). At the least self-determined end of the continuum is amotivation, defined by the absence of both autonomous and controlled motivations to engage in PE teaching (7).

For pre-service PE teachers, the process of perceiving how the teaching profession satisfies or frustrates their needs for autonomy and relatedness can often be complex. For instance, envisioning the extent of decision-making freedom or the quality of interactions with colleagues and students may be challenging, as it requires direct teaching experience. Nevertheless, after completing mandatory practicum periods (e.g., in Spain, a minimum of 6 weeks), pre-service teachers can better project how skilled and effective they feel (i.e., competence need) in teaching PE. Previous research suggests that competence satisfaction during PETE programs has predicted autonomous motivation for teaching PE, which, in turn, fosters the intention to implement autonomy-supportive (i.e., participative and attuning) and structuring (i.e., guiding and clarifying) teaching strategies. In contrast, competence frustration during PETE is linked to increased controlled motivation or amotivation, which makes adopting controlling (i.e., demanding and domineering) and chaotic (i.e., abandoning and awaiting) teaching strategies more likely (14, 24). Consequently, it seems essential for pre-service PE teachers to receive training during PETE on how to teach and interact with students in ways that enhance their sense of competence. This, in turn, can promote a more self-determined motivation for teaching, enhancing their intention to implement motivating teaching approaches (i.e., participative, attuning, guiding, and clarifying) while also reducing the use of demotivating approaches (i.e., demanding, domineering, abandoning, and awaiting) (24).

A systematic review by Reeve and Cheon (10) demonstrated that in-service PE teachers can learn and effectively implement autonomy-supportive teaching strategies. While most training programs have focused on autonomy support (i.e., participative and attuning approaches), recent research has also highlighted the importance of structuring strategies (i.e., guiding and clarifying) and, to a lesser extent, the role of controlling (i.e., demanding and domineering) and chaotic (i.e., abandoning and awaiting) teaching approaches (10). Several studies have shown that combining autonomy support with structuring strategies enhances student motivation and learning outcomes. Teachers who integrate clear expectations and constructive feedback within an autonomy-supportive framework promote greater BPN satisfaction, self-determined motivation, and engagement (25–27). Beyond autonomy-support and structuring teaching approaches, interventions aimed at reducing controlling teaching approaches have also improved student motivation and classroom climate (28, 29). A more comprehensive approach is seen in the training program by García-Cazorla et al. (30), which was the first to integrate the circumplex model into PE teacher training. Unlike previous programs that focused primarily on autonomy support, this initiative targeted all eight (de)motivating teaching approaches, providing a holistic framework to enhance motivating styles while reducing demotivating ones.

Regarding pre-service teachers, research on the design, implementation, and outcomes of intervention programs aimed at improving (de)motivating teaching styles remains scarce. Similar to the training of in-service teachers, existing programs for pre-service teachers have primarily focused on promoting autonomy support (or even reducing control), with less attention paid to structuring and chaotic styles. For example, Perlman (11) conducted a randomized controlled trial featuring a two-week online training program for pre-service PE teachers. The program yielded positive results, enhancing pre-service teachers’ ability to support student autonomy, reducing their reliance on controlling teaching styles, and ultimately enhancing students’ quality of motivation during their practicum lessons. Similarly, Großmann et al. (13) implemented a face-to-face intervention with pre-service biology teachers, focusing on understanding and applying autonomy-supportive teaching in future practice. The intervention revealed positive outcomes, enhancing both their knowledge of autonomy-supportive teaching styles and their intention to apply them in their future teaching. In both training programs with pre-service teachers, the intervention was the same for all participants (11, 13). However, tailoring PETE programs to individual needs seems crucial, as each pre-service teacher possesses distinct characteristics (e.g., age, gender) and a unique motivational background, leading to diverse teaching profiles and varying combinations of teaching approaches (14, 24). Therefore, PETE programs should adopt more personalized training methods that allow pre-service teachers to refine their motivating approaches while reducing their reliance on demotivating strategies.

For this purpose, observational methodologies such as video-analysis tools are crucial for delivering constructive and personalized feedback, as they allow pre-service teachers to objectively review their (de)motivating teaching styles, identify specific strengths and weaknesses, and engage in self-regulated learning (31). Additionally, motivational training programs should incorporate a structured theoretical phase, where pre-service teachers are introduced to SDT and the circumplex model, followed by practical sessions that closely simulate real-life teaching experiences. A particularly effective method is microteaching, in which pre-service teachers receive targeted feedback on their teaching approaches, allowing them to refine their strategies through iterative practice (11, 32). This is especially important because pre-service teachers often lack prior teaching experience, making experiential learning a crucial component of their training (24).

Furthermore, Reeve and Cheon (33) emphasize that teacher training should not only focus on instructional teaching styles but also on perspective-taking, helping educators understand students’ psychological needs before applying motivational strategies. Similarly, research suggests that the effectiveness of training programs is enhanced when the trainers themselves adopt a congruent teaching style. When trainers model motivating teaching strategies and avoid demotivating ones, pre-service teachers understand and appreciate the value of these pedagogical practices better (32). This underscores the importance of aligning theoretical training with practical demonstrations to maximize the impact of PETE interventions.

Building on the demonstrated benefits of SDT-based training programs in enhancing teachers’ motivating teaching styles, previous research has shown that these interventions contribute to greater student need satisfaction, enhance motivation for PE, improve well-being and engagement with the subject, and tend to strengthen students’ intention to participate in physical activity outside of school (10). Despite these positive outcomes, similar training initiatives remain scarce within PETE. To address this gap, the present mixed-method study outlines the protocol of a PETE-integrated training program grounded in SDT and the circumplex model. The program is designed to equip pre-service PE teachers with the knowledge and skills to implement motivating teaching approaches while minimizing demotivating teaching approaches. It is hypothesized that pre-service teachers will: (H1) perceive the training program positively; (H2) show an increase in the use of motivating teaching approaches (i.e., participative, attuning, guiding, and clarifying) alongside a decrease in demotivating teaching approaches (i.e., demanding, domineering, abandoning, and awaiting); and (H3) enhance their perceived competence, which, in turn, will foster greater autonomous motivation and decreased controlled motivation and amotivation for teaching PE.

## Method

### Context, design, and randomization

This study will be conducted at the University of Zaragoza in Spain, within the framework of the nationally standardized 60-credit Master’s degree in PETE, after obtaining a Bachelor’s degree in Physical Activity and Sport Sciences. This Master’s degree is a mandatory qualification for individuals aspiring to teach PE in secondary schools across Spain. The Máster’s degree program is structured into two distinct semesters. The first semester (i.e., September–January) focuses on theoretical training, covering pedagogy and curriculum design. The second semester (i.e., January–June) emphasizes practical application in PE contexts. The program concludes with a 7-week practicum where pre-service teachers gain hands-on teaching experience in secondary schools.

Due to the structural configuration of the Master’s degree program, random assignment to experimental and control conditions is not feasible. Implementing randomization within a compulsory academic subject would raise ethical concerns by restricting access to a pedagogical intervention that may benefit participants’ professional development. Moreover, logistical constraints such as fixed group assignments, centralized scheduling, and institutional regulations hinder randomization. Therefore, this study will employ a quasi-experimental pre-post design, incorporating an experimental group and a control group. They will undergo a three-phase assessment: pre-test, intermediate-test (only for the experimental group), and post-test. The study follows a mixed-methods approach, integrating quantitative and qualitative analyses to capture the intervention’s measurable outcomes and contextual nuances.

**Figure 1:**
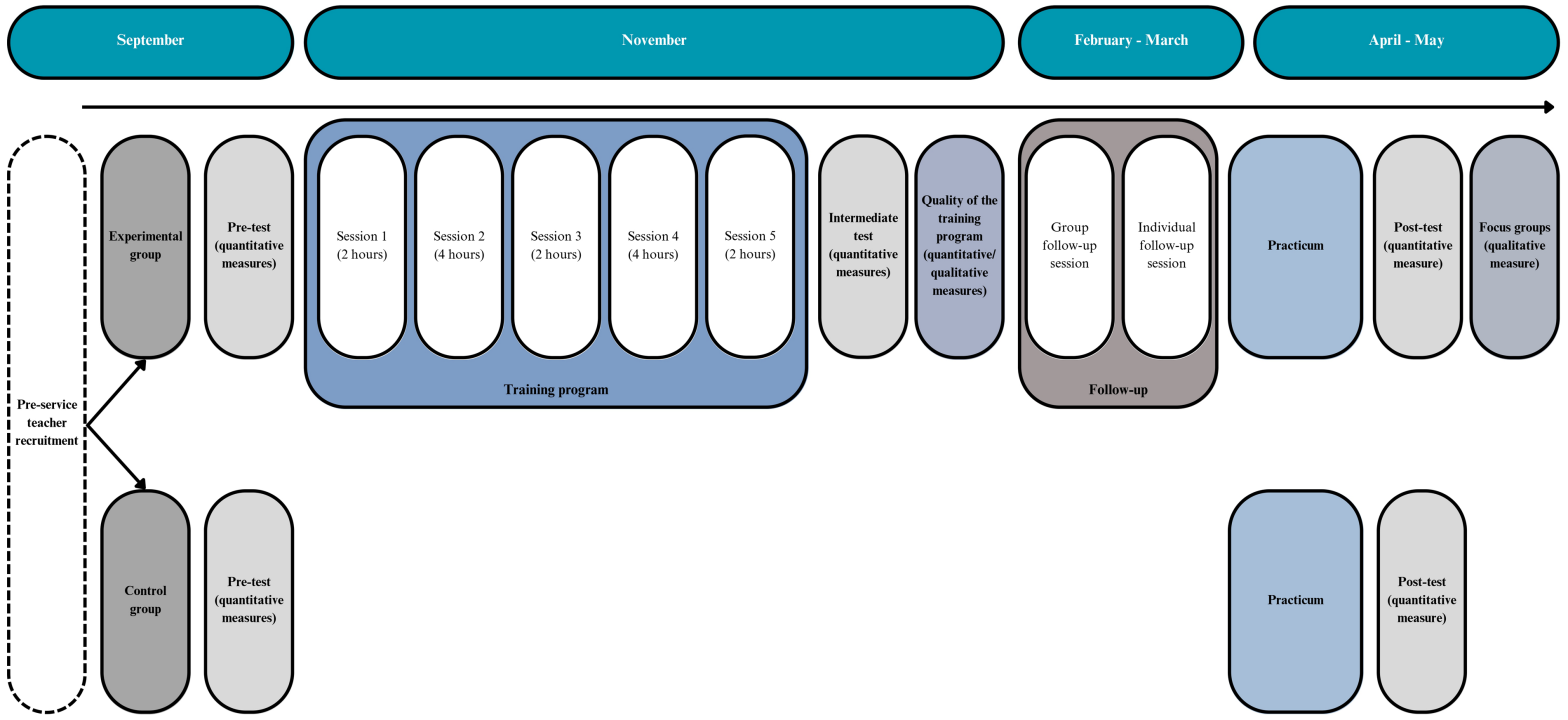
Characteristics of the training program and timeline for data collection.

Given the institutional constraints of the Master’s degree and the intention to integrate the training program within Master’s in PETE, the training will be delivered by university-affiliated faculty members with extensive experience in SDT and the circumplex model. The training program aligns with the progressive structure of the Master’s degree. During the first semester, pre-service PE teachers in the experimental group will receive theoretical and practical training based on SDT and the circumplex model. This phase will establish foundational knowledge of (de)motivating teaching strategies and their effects on student motivation and engagement. In the second semester, participants will apply this knowledge by designing PE lesson plans that integrate these strategies and will implement them during their practicum. Meanwhile, pre-service PE teachers in the control group will follow the standard Master’s curriculum without exposure to the intervention (see Figure 1). More details about the training program’s structure, content, and implementation can be found in the “Pre-service PE teachers’ training program” section.

The study was approved by the Ethics Committee of the University of Zaragoza.

### Sample size calculation

The sample size for this quasi-experimental protocol study was calculated using *R Studio* to ensure sufficient statistical power for detecting significant effects of the intervention. The calculation was based on a repeated-measures design, assuming a large effect size (*d* = 0.8), an 80% statistical power, and a significance level (*α* = 0.05), aligning with standard practices in educational research (34). To account for the inherent variability and potential selection biases associated with quasi-experimental designs, a 20% adjustment was applied to the initial estimate (35). Additionally, a 10% allowance for anticipated participant dropout was incorporated over the study period. After these adjustments, the final target sample size was set at 19 participants per group (i.e., 38 in total). This ensures adequate statistical power to detect meaningful differences while mitigating the limitations posed by participant attrition and the study design.

### Participants and recruitment

A minimum of 38 pre-service PE teachers (19 in the experimental group and 19 in the control group) will participate in the study. Participants will be selected using a non-randomized convenience sampling procedure, which is considered the most suitable approach given the structural and ethical constraints of the Master’s program. Specifically, the program includes only one cohort of students per academic year, making it logistically unfeasible to divide a single class into experimental and control conditions. Furthermore, assigning the intervention to only a subset of students within the same cohort would be ethically inappropriate, as it would create unequal access to pedagogical training aimed at enhancing professional competence. To prevent such disparities and avoid contamination between conditions, the control group will be recruited from one academic year and the experimental group from the following year.

While participation in the training program will be mandatory for students in the experimental group (as it is embedded within the Master’s in PETE), data collection will remain voluntary and anonymous. However, participants must meet specific inclusion criteria to be included in the study: (1) attending 100% of the training program sessions, (2) completing study questionnaires three times (i.e., pre-test, intermediate-test, and post-test), and (3) participating in a focus group at the end of the study.

### Measures

#### Questionnaires

The following variables of pre-service PE teachers will be measured through Google Forms at three time points: before the training program (T1; pre-test—at the beginning of the Master’s in PETE), during the program (T2; intermediate test—at the end of the first part of the training program), and after completing the program (T3; post-test—at the end of the Master’s in PETE practicum) (see Figure 1).

##### Socio-demographic variables

Age and gender will be self-reported by pre-service PE teachers.

##### Competence satisfaction and frustration toward PE teaching

The Basic Psychological Need Satisfaction and Frustration Scale (BPNSFS) (36) will be used to assess pre-service PE teachers’ self-perceived teaching competence. Starting with the phrase *“As a future PE teacher…,”* the four items measuring competence satisfaction (e.g., “I feel competent as a PE teacher”) and the four items measuring competence frustration (e.g., “I have serious doubts about whether I can do things well as a PE teacher”) will be included. Participants will respond using a five-point Likert scale ranging from 1 (*strongly disagree*) to 5 (*strongly agree*).

##### Motivation to teach

The Spanish version of the Motivation Scale for Teaching in Secondary Education (EME-ES) (37), adapted to the PE teaching context, will be used to assess pre-service PE teachers’ self-perceived motivation to teach. This scale begins with the prompt: *“I get involved in teaching Physical Education because.”* followed by 19 items that measure various forms of motivation. Specifically, it includes intrinsic motivation (4 items, e.g., “Teaching is fun”), identified regulation (4 items, e.g., “Teaching helps me learn new things”), introjected regulation (4 items, e.g., “I want to give others the impression that I am a good teacher”), external regulation (4 items, e.g., “It is assumed that I should do this”), and amotivation (3 items, e.g., “I do not know why I am a PE teacher, it is a useless job”). Participants will respond using a five-point Likert scale ranging from 1 (*strongly disagree*) to 5 (*strongly agree*).

##### (De)motivating teaching styles and approaches

The Spanish version of the Situations in School Questionnaire-Physical Education (SIS-PE) (14) will be used to evaluate pre-service PE teachers’ perception of their (de)motivating teaching approaches. The SIS-PE comprises 12 typical teaching situations consisting of four items each (i.e., 48 items in total). The 48 items are categorized according to the four overarching teaching styles. Autonomy-supportive style includes participative (four items) and attuning approaches (eight items). Structuring approaches comprise guiding (seven items) and clarifying approaches (five items). Controlling style is divided into demanding (seven items) and domineering approaches (five items). Chaotic style encompasses abandoning (eight items) and awaiting approaches (four items). An example of a situation is: *“In preparing for your class, you develop a lesson plan. Your priority is to..”* with four ways of answering: (1) “Offer challenges to the best students and provide sufficient support to exceptional students throughout their learning” (i.e., guiding approach); (2) “Do not plan the lesson too much. It will unfold on its own” (i.e., awaiting approach); (3) “Propose exercises that are pleasant, interesting, or very attractive” (i.e., attuning approach); (4) “Propose a lesson plan for all students to follow. There are no exceptions or excuses” (i.e., demanding approach). Participants will respond using a seven-point Likert scale, ranging from 1 (*It does not describe me at all*) to 7 (*It describes me perfectly*).

#### Quality of the training program

In line with previous intervention studies on both in-service (30, 32, 38) and pre-service PE teachers (13), the quality of the training program will be assessed at the end of the first phase (T2). For this purpose, the experimental group will complete a Google Forms questionnaire evaluating four key aspects of the training program: (1) the applicability of the acquired knowledge in real teaching contexts; (2) the alignment of the intervention program with their personal and professional interests; (3) the perceived usefulness of the content in their future teaching practice; and (4) the scalability of the training program for their long-term professional development. Participants will respond using a Likert scale ranging from 1 (*strongly disagree*) to 5 (*strongly agree*). Additionally, they must justify their responses through open-ended comments, providing qualitative insights into their perceptions of the training.

#### Focus groups

A focus group will be conducted at the end of the study, immediately after completing the post-test questionnaires (T3). The primary objective of this session is to gain deeper insights into pre-service PE teachers’ perceptions of the intervention program. The discussion will focus on three key areas: (1) their experiences related to changes in their (de)motivating teaching styles, motivation for teaching, and perceived competence throughout the program; (2) the perceived applicability of the strategies learned and their feasibility in real-world PE teaching contexts; and (3) the challenges encountered when implementing these strategies during their practicum. A general assessment of the strengths and weaknesses of the training program will also be made.

The focus group will be moderated by an expert in PE teaching instruction, the SDT framework, and qualitative methodology. To foster open and honest discussions among pre-service PE teachers, the trainers will not be present during the session. The discussion will follow a semi-structured format, ensuring a balance between guided questions and participants’ spontaneous contributions (see Table 1). The session will begin with a brief introduction, outlining the study’s objectives and procedures. The moderator will lead the discussion, supported by a co-moderator responsible for managing logistics, taking notes, and overseeing the recording equipment. At the end of the session, the co-moderator will summarize the key discussion points and invite participants to confirm the accuracy of the summary or provide additional insights. The focus group will be conducted in a comfortable and neutral setting, lasting approximately 50 min. All sessions will be videotaped and transcribed for in-depth analysis.

**Table 1:** Key questions for the focus group discussion.

Areas	Topic	Questions
Personal experiences	Changes in (de)motivating teaching styles	How do you think the training program influenced your style of teaching and interacting with students? Can you describe any specific changes in your teaching style?
	Changes in motivation for teaching	Has the training program affected your motivation for teaching PE? If so, in what ways?
	Perceived competence development	How has your sense of competence as a future PE teacher evolved throughout the program? Have there been specific moments that strengthened or challenged your confidence?
Applicability	Feasibility in real-world PE contexts	To what extent do you think the strategies learned in the training are feasible for implementation in real-world PE lessons? What factors facilitated or hindered their application?
Perceived challenges	Challenges during the practicum	What difficulties did you encounter when trying to apply the training content during your practicum? How did you address these challenges?
General feedback of the training program	Long-term impact on teaching practices	How do you think this training will influence your future teaching styles? Are there specific aspects of the program that you believe will have a lasting impact?
	Program strengths and areas for improvement	What aspects of the training program did you find most valuable? What modifications or improvements would you suggest for future editions of the program?

#### Ensuring objectivity and minimizing bias in the evaluation process

To reduce potential biases associated with the trainers also being evaluators, the assessment of pre-service teachers’ competence, motivation to teach, and (de)motivating teaching styles will be conducted by independent researchers who are not involved in delivering the training. Additionally, the focus group sessions will be moderated by an external expert in PE teacher education and SDT, ensuring that participants feel free to share their experiences without influence from their trainers. The trainers will not participate in these sessions or have access to individual responses until after the data collection phase is completed. This approach aims to enhance the objectivity of the evaluation and minimize the potential influence of social desirability biases in participants’ responses.

### Pre-service PE teachers’ training program

#### Experimental group

**Figure 2:**
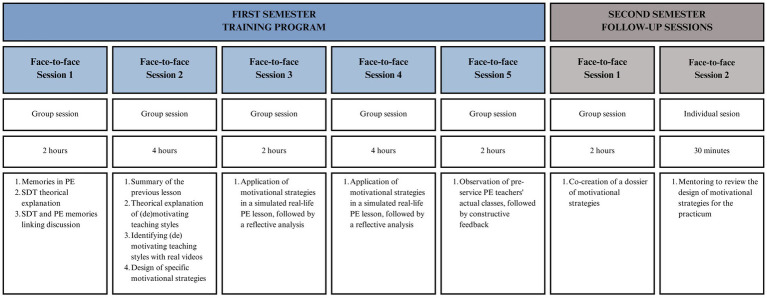
Summary of the training program sessions.

The intervention for the experimental group will consist of two phases: (1) a teacher-training phase, comprising five face-to-face sessions, and (2) a follow-up phase, where pre-service PE teachers will design and apply SDT-based strategies during their Master’s practicum (see Figure 2).

The first phase of the training program will take place within the Master’s in PETE subject titled “Curriculum and Instructional Design in Physical Education,” scheduled during the first semester (i.e., September–January) in the first 3 weeks of November as part of the instructional design module. The first phase of the program consists of 14 face-to-face hours over 3 weeks, with sessions structured as follows: Tuesdays (16:00–18:00, two-hour sessions) and Wednesdays (15:00–19:00, four-hour sessions) in the first 2 weeks, concluding with a final two-hour session on Tuesday (16:00–18:00) in the third week. The primary aim of this phase is to enhance pre-service PE teachers’ autonomy-supportive and structuring teaching styles while reducing controlling and chaotic styles, following SDT and the circumplex model. Two university teachers with expertise in SDT-based training programs for PE teachers will lead the sessions. It is essential that trainers model a congruent teaching style (32), adopting motivating instructional strategies that support autonomy and structure while avoiding controlling and chaotic styles.

The first week of the program will start with the first two-hour face-to-face session, which will be primarily theoretical (32, 38). It will begin with a brief introduction by the trainers, outlining the program structure and objectives. Following this, the session will start with an interactive activity (15 min) called “Memories in PE.” In this activity, pre-service teachers will autonomously write one positive memory of their PE teacher’s behavior on a green sticky note (e.g., “They helped us with any problem we had”) and one negative memory on a red sticky note (e.g., “They made us do exercises exactly as they instructed, or we were punished”). Once completed, participants will place their sticky notes on the classroom whiteboard for discussion at the end of the session. This activity serves as an experiential bridge between their past experiences and the following theoretical content, helping to personalize and contextualize the learning process. With this reflective foundation, the trainers will proceed with the theoretical training based on SDT, focusing specifically on the role of BPN in motivation (70 min). To foster engagement, trainers will actively involve participants through guided questions (e.g., “What do you understand by BPN?”) and open discussions (e.g., “What are the differences between autonomy and competence?”). At the end of the session, trainers will read aloud the “Memories in PE” responses, initiating a group discussion on how these experiences align with BPN and their impact on motivation in PE (30 min). The session will conclude with a brief explanation of an individual assignment. Each pre-service teacher will record a short (maximum 4 min) video explaining the SDT motivational sequence. Additionally, a brief explanation of the next steps and the objectives for upcoming sessions will be given to foster a positive disposition among the teachers (5 min).

The second session of the program, lasting 4 h, will adopt a theoretical-practical approach. In the first part of the session, a brief review of SDT from the previous session will be conducted. Additionally, the (de)motivating teaching styles proposed by the circumplex model will be introduced, explaining how these styles influence students’ BPN and their motivation in PE (100 min). After this theoretical segment, participants will have a 20-min break before transitioning into the practical part. In the second part of the session, pre-service teachers will be divided into small working groups of 4–5 participants. Each group will analyze a series of video clips showcasing different (de)motivating teaching styles in authentic PE lessons. Their primary task will be to identify and categorize the different (de)motivating styles observed in the videos. If there are any doubts, the corresponding videos will be presented to the entire group, allowing the trainers and participants to collectively analyze the teaching styles and reflect on their consequences for students’ BPN and motivation (60 min). Finally, pre-service teachers will regroup, and each group will select a PE content area to design a lesson plan incorporating motivational strategies that support students’ BPN. Trainers will actively supervise and provide formative feedback, addressing questions and guiding participants in refining their lesson plans (55 min). To conclude the session, trainers will introduce the next phase of the training program. They will explain that the upcoming sessions will be practical, where pre-service teachers will implement the lesson plans they developed in this session. These simulated lessons will be conducted with their peers acting as secondary school students, providing an opportunity for hands-on practice and feedback.

The second week of the training program will commence with the third face-to-face session, which will be fully practical and will span 2 h. Through a random selection process, two members from each group will be chosen to deliver the PE lesson they previously designed. One will lead the first half of the session, and the other will take over for the second half. Each teaching pair will have 45 min to implement their lesson, during which their peers will assume the role of secondary school students, simulating a real classroom environment. Additionally, a separate observer group consisting of 4–5 peers will use a structured rubric to systematically assess and record the (de)motivating teaching styles and instructional strategies employed by the pre-service teachers who were acting as instructors at that time. After each lesson, the observer group will rotate, allowing a new set of participants to assume the observer role, ensuring that all pre-service teachers experience both teaching and observational perspectives. Following each session, the observers and trainers will engage in a guided reflective discussion, providing constructive feedback on the teaching strategies used, their alignment with SDT principles, and their effectiveness in supporting students’ BPN (10 min). Given the two-hour duration, two sessions can be implemented at this time. To further enhance learning, each session will be video recorded. These recordings will be revisited in the final session of the training program, allowing participants to engage in self-reflection, peer review, and deeper analysis of their teaching styles and areas for improvement.

The fourth face-to-face session of the first phase of the training program will be a practical session, following the same structure as the third session but with an extended duration of 4 h instead of two. Each group will have 45 min to implement their lesson, followed by a 10-min feedback period, during which trainers and the assigned observer group will provide reflections on the session and the motivational strategies employed. The session will begin with the first two teaching implementations, followed by a 15-min break, after which the remaining two sessions will be conducted. This structure ensures that in each group, two members (i.e., pre-service teachers) will have the opportunity to teach, while all participants will rotate through the roles of students and observers. As in the previous session, the entire session will be recorded for later review and analysis in the final session of the training program.

To conclude the first phase of the training program, a final two-hour face-to-face session will be held during the third week. This session will involve presenting and analyzing selected excerpts from the recorded videos of the previous two practical sessions, highlighting both motivating and demotivating teaching strategies. The primary goal of this session is to stimulate critical discussion, enabling pre-service teachers to analyze their own and their peers’ teaching strategies. Participants will be encouraged to identify areas for improvement and propose alternative strategies. Trainers will moderate the discussion, guiding participants toward recognizing and replacing demotivating styles with strategies that better support students’ BPN.

The follow-up phase of the training program will take place in February and March as part of the subject “Design of physical education learning activities,” which is conducted during the second semester (i.e., January–June) of the Master’s in PETE. During this subject, pre-service teachers are tasked with designing a teaching unit that they will later implement during their practicum. Accordingly, the second phase of the program comprises two in-person sessions. The first session, scheduled for the third week of February and lasting 2 h, will serve as a review workshop. During this session, pre-service teachers will work in small groups to develop motivational strategies and share their proposals with their peers. This collaborative process will facilitate the co-creation of a dossier with motivational strategies, which will serve as a reference to support them in designing the sessions of their teaching unit.

The second session, set for the second week of March, will consist of individual face-to-face mentoring meetings. In these sessions, trainers will review the teaching unit sessions for each pre-service PE teacher. Each mentoring session will last approximately 30 min. The main objective is to review the lesson plans and designed motivational strategies, provide tailored guidance, and address any questions prior to one of the most critical phases of the Master’s in PETE, the practicum.

#### Control group

Participants assigned to the control group will not receive the structured training program designed for the experimental group. Instead, they will follow the standard curriculum of the Master’s in PETE, which includes general instruction on teaching skills and motivation in PE. While this training also incorporates elements of SDT, it is delivered in a more theoretical and lecture-based format, without the applied, interactive, and iterative components present in the experimental group’s intervention. Additionally, although the total number of instructional hours dedicated to teaching skills is comparable between both groups, the control group’s training does not specifically focus on the circumplex model or include practical components such as microteaching, video analysis, or structured feedback. Their participation in the study will be limited to completing the questionnaires at two specific time points: before starting the Master’s (i.e., pre-test) and upon its completion (i.e., post-test).

### Analysis plan

#### Quantitative analyses

Firstly, to ensure statistical assumptions are met, Levene’s test will be used to assess the homogeneity of variances, while the Kolmogorov–Smirnov test will verify the normality of the data distribution (*p* > 0.05). Additionally, Cronbach’s alpha will be computed for each study variable across the three measurement points to evaluate the internal consistency reliability. To analyze the effects of the intervention a 3 × 2 (Time × Condition) repeated-measures multivariate analysis of covariance (MANCOVA) will be conducted, including pre-service teachers’ gender as a covariate. Consequently, this approach will allow for the assessment of both the main effects of the intervention and potential interactions with gender, providing a more precise understanding of its impact on different teaching profiles. In addition, multiple paired t-tests with Bonferroni correction will be performed to assess differences between groups (i.e., experimental vs. control) as well as within groups (i.e., pre-test vs. post-test). Effect sizes will be interpreted following Cohen’s criteria, considering values of 0.01 as small, 0.06 as moderate, and 0.14 as large. All statistical analyses will be carried out using IBM SPSS Statistics v.29.0. Moreover, a longitudinal structural equation model will be employed to examine the predictive relationships between the study variables, enabling the assessment of potential changes across the three measurement points (i.e., pre-test, intermediate-test, and post-test).

#### Qualitative analyses

Qualitative data from the focus groups will be transcribed and analyzed using NVivo 11.0, following the thematic analysis framework outlined by Braun and Clarke (39). Initially, three researchers will independently review all transcripts to become thoroughly familiar with the data. Next, they will code, identify and extract segments of text that pertain to pre-service teachers’ perceptions of the training program’s effects and their experiences applying strategies acquired during the practicum. Themes will be generated based on the most salient and recurrent meanings within the dataset. Given the alignment between the focus group questions and the study’s theoretical framework, a deductive thematic analysis will be conducted, informed by SDT and the circumplex model. To enhance analytical rigor, two additional researchers will oversee the coding process, contributing alternative interpretations and supporting consensus-building, thereby increasing inter-rater reliability. Finally, where appropriate, qualitative findings will be triangulated with quantitative data to enrich interpretation and offer a more comprehensive understanding of the program’s effects.

## Discussion

Interventions grounded on SDT have demonstrated effectiveness in enhancing the motivating teaching styles of in-service PE teachers. However, replicating these interventions remains challenging, as many protocols are not always reported in detail. Furthermore, research on training programs targeting pre-service teachers is still scarce, despite the crucial role in shaping future pedagogical practices. This challenge becomes even greater in university-based initial teacher education programs, where transferring successful interventions to future teachers is particularly complex, owing to the difficulties in applying theoretical knowledge in practical settings. In response to this gap, the present study protocol outlines an SDT- and the circumplex model-based intervention designed to improve autonomy-supportive and structuring approaches (i.e., participative, attuning, guiding, and clarifying) while reducing controlling and chaotic approaches (i.e., demanding, domineering, abandoning, and awaiting) in pre-service PE teachers.

This study is expected to provide key contributions to the literature on PETE: (1) it pioneers a motivational training program based on the circumplex model tailored for pre-service PE teachers, addressing an existing gap in initial teacher education by focusing on both motivating and demotivating teaching approaches; (2) the training’s effectiveness will be assessed using a multi-method evaluation strategy, integrating both a training-end questionnaire and a concluding focus group, thus capturing participants’ immediate and reflective insights; (3) a robust mixed-methods approach will be employed, integrating quantitative measures (i.e., validated questionnaires) and qualitative insights (i.e., focus group discussions) to explore changes in teaching styles, competence satisfaction, and motivation for teaching; (4) the study will explore potential gender-based differences in response to the training, examining potential variations in how male and female pre-service teachers experience and implement motivational strategies. It aims to provide nuanced insights that could guide the development of more tailored PETE interventions; (5) by incorporating multiple assessment points (i.e., pre-test, intermediate-test, and post-test), the study will capture the progress of participants’ teaching approaches, rather than relying solely on pre-post comparisons; (6) embedding the training program within the established Master’s in PETE curriculum ensures that the intervention is both contextually relevant and scalable for broader application in teacher education; and (7) the intervention will employ research-backed methodologies, including congruent teaching, video analysis of real teaching scenarios, microteaching exercises, collaborative development of teaching strategies, and personalized mentoring, facilitating meaningful integration of motivating teaching styles into pre-service PE teachers (30–32, 38).

The expected outcomes of this training program for pre-service PE teachers will be analyzed in light of the study’s three hypotheses. Regarding the first hypothesis (H1), as previously validated strategies from SDT-based training programs will be incorporated (i.e., BPN-awareness, microteaching, video analysis, co-creation of teaching strategies, and expert mentoring) (32, 33, 38) it is expected that pre-service PE teachers in the experimental group will positively evaluate the training program, perceiving it as useful and applicable to their future professional practice. Their feedback will be instrumental in refining the program to enhance its acceptability, sustainability, and scalability, ensuring its feasibility for implementation in other PETE programs.

Regarding the second hypothesis (H2) and given that (de)motivating teaching styles are teachable, malleable, and learnable (10, 40), it is expected that pre-service PE teachers in the experimental group will demonstrate a significant increase in the use of autonomy-supportive (i.e., participative and attuning) and structuring (i.e., guiding and clarifying) teaching styles. Simultaneously, a reduction in controlling (i.e., demanding and domineering) and chaotic (i.e., abandoning and awaiting) teaching styles is anticipated. However, it is important to acknowledge that, as pre-service teachers with no prior teaching experience, they may not perceive or report changes in their teaching styles, as observed in the study by Perlman (11). This underscores the importance of incorporating observational and reflective methodologies, such as video analysis and peer feedback, to depict behavioral changes beyond self-reported perceptions accurately.

Finally, concerning the third hypothesis (H3), pre-service teachers are expected to develop a stronger sense of competence in their instructional abilities by expanding their repertoire of motivational teaching strategies. This, in turn, should lead to higher competence satisfaction and lower competence frustration throughout their training. Following the motivational sequence proposed by SDT, it is anticipated that enhanced perceived competence will foster higher levels of autonomous motivation for teaching PE, while simultaneously reducing controlled motivation and amotivation (24). These changes not only have implications for pre-service teachers’ immediate development but may also contribute to long-term professional engagement and teaching quality once they enter the workforce.

## Limitations

This study has several limitations that should be acknowledged. First, the quasi-experimental design prevents random assignment, potentially introducing selection bias and precluding causal inferences. However, implementing a fully randomized controlled trial in an educational context presents both practical and ethical challenges. Given that the training program is embedded within an official teacher education curriculum, it would be unfeasible and arguably unethical to randomly assign pre-service teachers to receive or be denied pedagogical training that could enhance their professional development. Restricting access to an evidence-based intervention could disadvantage certain participants and create inequalities in their preparation for future teaching. Moreover, logistical constraints, such as fixed course enrollments and institutional policies, further limit the feasibility of random allocation. Second, this study is conducted within a single Spanish university, which restricts the generalizability of its findings. To improve the external validity, future studies should expand the sample across multiple universities, both nationally and internationally, ensuring greater cross-context applicability of the training program. Third, the study primarily focuses on short-term effects, assessing pre-service teachers’ (de)motivating teaching styles during training and practicum. However, it remains unclear whether these effects will persist once they transition to full-time teaching positions. To address this issue, longitudinal follow-ups should be conducted to examine the sustainability and long-term impact of the intervention. Fourth, the integration of the training program into the Master’s in PETE presents a logistical challenge, as the limited time available within the program constrains the full implementation of all the training components without disrupting other coursework. However, rather than modifying the program’s structure, a more impactful approach may be to advocate for its institutionalization within teacher education curricula. If the training proves effective, collaborating with policymakers and educational stakeholders could help establish it as a standardized component of PETE. This would ensure that all pre-service PE teachers receive systematic training in motivational teaching strategies without compromising other essential aspects of their teacher education. Fifth, assessing actual changes in teaching behaviors remains complex, as the study relies on self-reported data rather than direct observation of classroom practices. To strengthen data triangulation, future research should integrate student-reported measures and independent classroom observations by external evaluators. Finally, although the study includes an observational phase during the practicum, its effectiveness may be compromised by external constraints. These constraints include mentor teachers imposing specific teaching methods, restricting instructional autonomy, or limiting pre-service teachers’ ability to apply the motivational strategies learned during training. Future studies should explore alternative practicum models where pre-service teachers have greater instructional freedom or collaborate with practicum supervisors to align expectations regarding teaching autonomy.

## Conclusion

This study presents a comprehensive protocol for a motivational training program designed to enhance pre-service PE teachers’ motivating teaching styles. By integrating SDT and the circumplex model within PETE, this program aims to promote the use of autonomy-supportive and structuring teaching styles, while reducing reliance on controlling and chaotic styles. In doing so, it seeks to foster a more need-supportive learning environment, ultimately benefiting both teachers’ pedagogical approaches and students’ motivational experiences in PE. By employing a quasi-experimental, mixed-methods design, it seeks to provide empirical evidence on how such training programs influence competence satisfaction, autonomous motivation for teaching, and the application of motivational strategies in both training and practicum settings. The structured nature of the intervention ensures that it is replicable and scalable, making it adaptable to various PETE contexts. If the expected outcomes are confirmed, the findings could significantly contribute to advancing evidence-based motivational training programs, offering a scientifically grounded framework for strengthening the preparation of future PE teachers. Furthermore, this research could serve as a foundation for future studies aimed at refining and expanding motivational interventions in teacher education, ultimately supporting the development of more effective, engaging, and student-centered PE instruction.
